# An Extremely Rare LAMA2 Gene Variant c.442C>T (p.Arg148Trp) Causing Late-Onset LAMA2-Related Dystrophy

**DOI:** 10.7759/cureus.61897

**Published:** 2024-06-07

**Authors:** Alvee Saluja, L. H Ghotekar, Shahbaz Anees, Anul Haque, Rajinder K Dhamija

**Affiliations:** 1 Neurology, Lady Hardinge Medical College, New Delhi, IND; 2 Internal Medicine, Lady Hardinge Medical College, New Delhi, IND; 3 Neurology, Institute of Human Behaviour and Allied Sciences, New Delhi, IND

**Keywords:** c.442c>t (p.arg148trp), limb-girdle muscle dystrophy, lama2, lama2-related muscular dystrophies, congenital muscular dystrophy

## Abstract

Mutations in the alpha-2 subunits of the laminin gene (LAMA2) cause an autosomal recessive congenital muscular dystrophy (CMD) subtype known as laminin a2-related muscular dystrophies (LAMA2-RD). LAMA2-RD can present with a wide range of phenotypes ranging from severe infantile congenital muscular dystrophy to milder adult-onset limb-girdle muscular dystrophy. This case describes a 28-year-old Indian gentleman having childhood-onset focal seizures, gradually progressive proximal predominant lower-limb weakness for the past three years, elevated creatinine phosphokinase levels, and MRI brain suggestive of diffuse symmetrical periventricular white matter hyperintensities. The whole exome sequencing revealed a rare homozygous missense variant in exon 4 of the LAMA2 gene on chromosome 6 (c.442C>T[p.Arg148Trp]). Adult-onset limb-girdle muscular dystrophy with white matter imaging abnormalities, hyperCKemia, and seizures should evoke suspicion of LAMA2-RD. This case brings forth an ultra-rare genetic mutation that has not been previously reported in individuals of South Asian ethnicity leading to LAMA2-RD. More cases of late-onset LAMA2-RD from various ethnicities need to be reported to expand our understanding of the clinical-genetic spectrum of the disease.

## Introduction

Congenital muscular dystrophies (CMD) are a group of rare disorders with a worldwide prevalence of 0.5-2.5 per 100,000 individuals [1-3]. CMDs typically present early in life (infancy or childhood) with developmental delay, hypotonia, and joint contractures and result from genetic mutations that cause either a partial or complete deficiency of structural skeletal muscle proteins. Depending on the location and type of the protein affected, CMDs are classified into a) basement membrane and extracellular matrix abnormalities, b) alpha-dystroglycan glycosylation defects, c) endoplasmic reticulum protein defects, d) nuclear envelope protein disturbances, e) Golgi apparatus and endoplasmic reticulum protein trafficking defects; and f) other CMDs/CMD-plus conditions (such as CMD with extrapyramidal or cerebellar involvement, Marinesco-Sjogren syndrome, etcetera) [3]. Mutations in the alpha-2 subunits of the Laminin (LAMA2) gene result in the deficiency of a key structural skeletal muscle protein known as merosin and cause an autosomal recessive congenital muscular dystrophy (CMD) subtype known as the laminin a2-related muscular dystrophies (LAMA2-RD). LAMA2-RD accounts for roughly 1/3rd of CMD cases with an estimated prevalence of 0.6-0.7/100,000 individuals [4]. LAMA2-RD has a wide range of phenotypes, from severe infantile to milder late-onset forms [5]. A young adult with progressive lower limb weakness due to a rare missense variant not previously reported in the South Asian population is described.

## Case presentation

A 28-year-old Indian gentleman born of a non-consanguineous marriage via a full-term vaginal delivery had normal milestones. At seven years of age, he developed focal seizures with impaired awareness. The neuroimaging done at that time showed periventricular white matter hyperintensities. Based on the neuroimaging, he was labeled as having a leukodystrophy and prescribed sodium valproate. His last seizure was five years ago. At 25, he started having difficulty getting up from a squatting position. For the past two years, he could not get up from the floor without support and required the help of the side railings to climb upstairs. The family history was unrevealing.

The higher mental functions were normal. On motor system examination, the hip extensors were weaker than the flexors (MRC power grade 3/5 vs. 4/5), and the adductors were weaker than the abductors (MRC power grade 3/5 vs. 4/5). Power in the knee flexors was MRC grade 4/5, and power at the ankle joint was 5/5. The deep tendon reflexes were normal. Hypertrophied extensor digitorum brevis and tendoachilles contractures were present bilaterally. The cranial nerve, sensory, cerebellar, and extrapyramidal system examinations were normal. The complete hemogram, liver and renal functions, fasting blood sugars, serum vitamin D3, B12, and thyroid function tests were normal. The human immunodeficiency virus, hepatitis B surface antigen, anti-hepatitis C virus antibody, and anti-nuclear antibodies were negative. The creatinine phosphokinase (CPK) level was 1324 IU/L (normal range, <171 IU/L) (Table 1).

**Table 1:** Results of the blood investigative workup of the patient Hb: Hemoglobin, TLC: Total leukocyte count, IU/L: International units/Litre, gm/dl: gram/decilitre, mg/dl: milligrams/decilitre, mEq/L: milliequivalents/Litre, pg/ml: picograms/millilitre, ng/dl: nanograms/decilitre, nmol/L: nanomole/litre, AST: Aspartate transaminase, ALT: Alanine transaminase, ALP: Alkaline phosphatase, TC: Total cholesterol, TG: Triglycerides, LDL: Low-density lipoprotein, HDL: High-density lipoprotein, HbA1c: Glycated hemoglobin, HIV: Human immunodeficiency virus, HBsAg: Hepatitis B surface antigen, HCV: Hepatitis C virus, ANA: Anti-nuclear antigen, RA: Rheumatoid antigen, c-ANCA: cytoplasmic antineutrophilic cytoplasmic antibody, p-ANCA: Perinuclear antineutrophilic cytoplasmic antibody, TSH: Thyroid stimulating hormone, fT3: free T3, fT4: free T4, LDH: Lactate dehydrogenase, CPK: Creatinine phosphokinase

Investigation	Result	Reference range
Hb	13.2 gm/dl	13-18 gm/dl
TLC	5640/microlitres	4000-10,000/microlitres
Platelet count	225 x 10^3^/microlitres	150-410 x 10^3^/microlitres
AST	45 IU/L	5-35 IU/L
ALT	30 IU/L	5-45 IU/L
ALP	74 IU/L	48-128 IU/L
T. Bilirubin	0.4 mg/dl	0.00-2.00 mg/dl
Urea	23 mg/dl	15-40 mg/dl
Creatinine	0.6 mg/dl	0.6-1.3 mg/dl
Sodium	139 mEq/L	136-145 mEq/L
Potassium	4.7 mEq/L	3.5-5.1 mEq/L
Calcium	8.6 mg/dl	8.6-10.2 mg/dl
Phosphate	3.0 mg/dl	2.5-4.5 mg/dl
TC	172 mg/dl	0-200 mg/dl
TG	150 mg/dl	0-150 mg/dl
LDL	127 mg/dl	0-130 mg/dl
HDL	41 mg/dl	40-60 mg/dl
HbA1c	5.5%	4-5.6%
Blood sugar-Fasting	91 mg/dl	70-100 mg/dl
HIV	Negative	Negative
HBsAg	Negative	Negative
IgM Anti-HCV antibody	Negative	Negative
ANA	Negative	Negative
RA factor	Negative	Negative
c-ANCA	Negative	Negative
p-ANCA	Negative	Negative
ENA profile	Negative	Negative
TSH	4.86 mIU/ml	0.34-5.6 mIU/ml
fT3	3.13 pg/ml	2.1-4.4 pg/ml
fT4	0.81 ng/dl	0.8-2.7 ng/dl
B12	374 pg/ml	180-914 pg/ml
Folic Acid	14.87 nmol/L	6.5-35 nmol/L
Vit. D3	16.82 ng/ml	30-70 ng/ml
LDH	245 IU/L	140-280 IU/L
CPK	1324 IU/L	<171 IU/L

**Figure 1:**
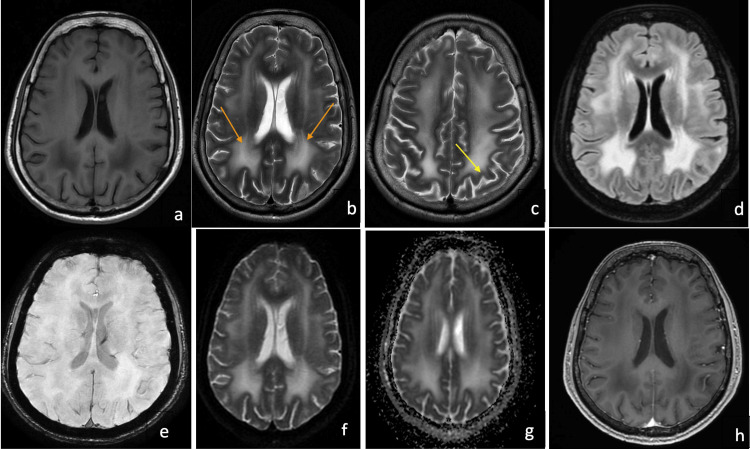
Magnetic resonance imaging (MRI) of the brain in the index case 1a. T1 weighted axial MRI of the brain showing bilateral symmetrical hypointensity in the periventricular white matter, 1b. T2 weighted axial MRI of the brain showing bilateral symmetrical hyperintensities in the periventricular white matter (orange arrows), 1c. T2 axial MRI brain highlighting the involvement of the subcortical U-fibers (yellow arrow), 1d. FLAIR hyperintensities noted in the bilateral periventricular white matter, 1e. Susceptibility-weighted imaging (SWI) showed no evidence of microbleeds, 1f. and 1g. Diffusion-weighted imaging (DWI) and attenuation diffusion coefficient (ADC) images demonstrate hyperintense signals in the periventricular white matter suggesting a T2 shine-through effect (probably due to vasogenic edema resulting from hyperpermeability of blood capillaries secondary to LAMA2 protein defect), 1h. T1 contrast images demonstrate the lack of contrast enhancement in the white matter lesions.

The nerve conduction studies did not show any evidence of demyelinating peripheral neuropathy and were normal. Needle electromyography suggested a myopathic pattern in the lower limbs. A biopsy from the right vastus lateralis showed extensive replacement of muscle with fibrofatty tissue, suggesting end-stage muscle disease. Immunohistochemistry (IHC) was inconclusive, and the presence or absence of merosin could not be determined due to the extensive fibrofatty replacement of the muscle fibers. Since there was no evidence of skin involvement (such as blistering or bullae formation), a skin biopsy was not carried out. The MRI brain confirmed diffuse symmetrical periventricular white matter hyperintensities and involvement of U-fibers (Figures 1a-1h). There was no evidence of cerebellar hypoplasia or occipital neuronal migration abnormalities on the MRI brain in this patient. 

**Figure 2:**
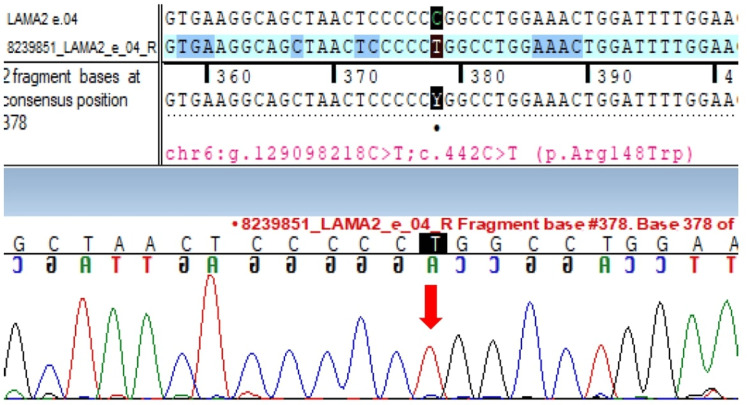
Sanger sequence chromatogram highlighting the homozygous LAMA2 mutation in the proband Sanger sequence chromatogram and alignment to the reference sequence showing the variant in exon 4 of the LAMA2 gene in the homozygous state in the proband (chr6:g.129098218C>T; c.442C>T; p.Arg148Trp) (indicated by the red arrow).

Whole exome sequencing revealed a homozygous missense variant in exon 4 of the LAMA2 gene on chromosome 6 (c.442C>T [p.Arg148Trp]) (Figure 2).

**Figure 3:**
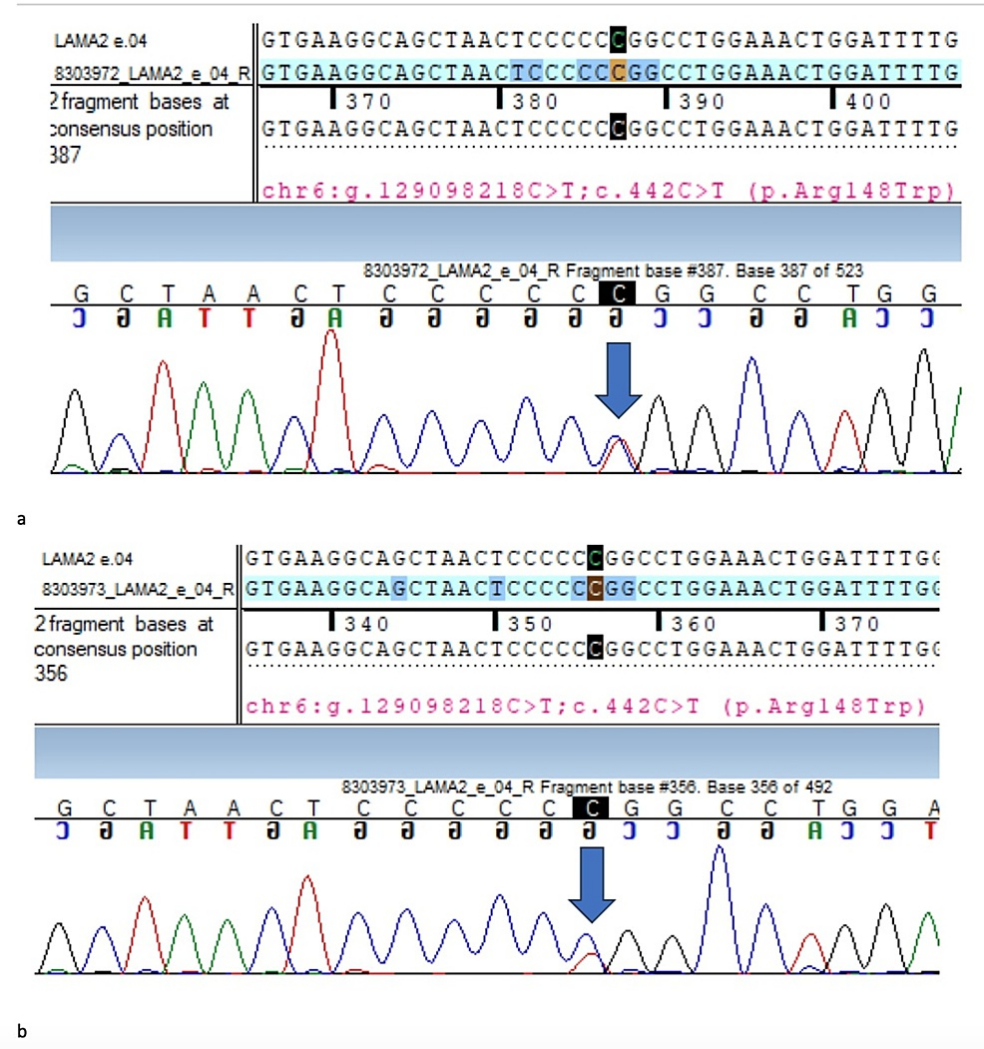
Sanger sequence chromatograms highlighting the LAMA2 status in the mother and the sister of the index case The Sanger sequence chromatogram and alignment to the reference sequence show the same variant in the heterozygous state in the asymptomatic mother (Figure 3a) and the sister (Figure 3b), suggesting a carrier state in both of them (indicated by blue arrows)

In-silico prediction analysis was damaged by PolyPhen-2 (HumDiv), SIFT, LRT, and MutationTaster2. Segregation analysis revealed the same variant in a heterozygous carrier state in the unaffected mother and sister (Figures 3a, 3b).

His father had expired and could not be tested. Thus, based on the clinical phenotype, radiological findings, and genetic analysis, he was diagnosed as a case of LAMA2-RD. He was advised regular physiotherapy exercises, sodium valproate, oral calcium, and vitamin D3 supplementation and is currently under follow-up.

## Discussion

LAMA2-RD classically presents within the first six months with severe hypotonia, axial weakness, delayed motor milestones, joint contractures, elevated creatinine phosphokinase levels, and white matter abnormalities on neuroimaging. Seizures can occur in up to 1/3rd of patients, are drug-responsive, and can be either generalized or focal [6,7]. Late-onset LAMA2-RD can present from childhood until adulthood as a milder CMD phenotype or limb-girdle muscle dystrophy [8]. Predicting the exact prevalence of this form is difficult due to its rarity and the heterogeneity in presentation [9]. The clinical phenotype of our case was consistent with the late-onset form of LAMA2-RD. The clinical presentation is affected by the genotype, and missense variants may be associated with late-onset disease [10].

The detected variant (c.442C>T [p.Arg148Trp]) substitutes tryptophan (non-polar) for arginine (polar) at codon 148 in the Laminin N-terminal domain of the LAMA2 protein, possibly altering the protein function. This variant has not been reported in the 1000 genome databases. The minor allele frequency was 0.00066%, 0.00119%, and 0.00076% in the gnomAD (v3.1), gnomdAD (v2.1), and TOPmed databases. The variant fulfilled two moderate (located in a well-established functional domain [PM1] and absent from controls in the 1000 Genomes Project [PM2]) and two supportive (multiple lines of computational evidence supporting deleterious effect on the gene or gene product [PP3] and highly specific patient phenotype for a disease with a single genetic etiology [PP4]) criteria for a likely pathogenic variant in this case [11]. This variant has only been reported once in a Sudanese family with congenital muscular dystrophy. In contrast to this case where motor symptoms (limb-girdle weakness) developed at 25, the previously reported case(s) with this mutation had presented with poor motor development and hypotonia at three and four years of age. Furthermore, muscle biopsy and MRI brain were not done in the previously reported case(s) (Table 2) [12].

**Table 2:** Comparison of the present case with the prior case report on c.442C>T ([p.Arg148Trp]) LAMA2 gene mutation CK: Creatinine kinase, MRI: Magnetic resonance imaging, IHC: Immunohistochemistry, LAMA2: alpha-2 subunit of laminin

Previous Case Report [Amin et al, 2019]^12^	Present Case
The onset of motor symptoms: Two siblings with the age of onset at 3 years (proband) and 4 years (sibling) were described. The symptoms were poor motor development (delayed motor milestones), hypotonia, and proximal muscle weakness. A history of epilepsy in the sibling was noted.	The onset of motor symptoms: A single case with a limb-girdle pattern of weakness beginning at 25 years of age. The only other symptom was seizures (noted in childhood at 7 years of age).
Ethnicity: African	Ethnicity: South Asian
History of consanguinity and family history: First-degree consanguinity was present and a similar history of motor symptoms in a maternal uncle who expired at 36 years of age was noted.	History of consanguinity and family history: Negative
CK levels: 2 x upper limit of normal	CK levels: 8 x upper limit of normal
Muscle biopsy and Immunohistochemistry: Not done	Muscle Biopsy and Immunohistochemistry: Extensive replacement of muscle with fibrofatty tissue suggesting end-stage muscle disease. The IHC was done but the results were inconclusive.
MRI Brain: Not reported/done	MRI Brain: Characteristic bilateral symmetrical hyperintensities in the periventricular white matter with involvement of the U-fibers noted.
Genetic confirmation: Sanger validation was done in the proband, parents, and a healthy sister. Sanger validation was not done in the affected sibling (as he had expired). Homozygous mutation LAMA2 gene c.442C>T (p.Arg148Trp) was detected in the proband. A carrier state was observed in the parents. A Wild-type gene was present in the healthy sister.	Genetic confirmation: Sanger validation was done in the proband, unaffected mother and sister. The father had expired and was not available for testing. LAMA2 c.442C>T (p.Arg148Trp) homozygous mutation was detected in the proband. A carrier state was observed in the unaffected mother and sister.

Thus, this case adds a late-onset limb-girdle dystrophy to the phenotypic spectrum of this extremely rare genetic mutation. The available evidence on novel genetic mutations causing LAMA2-RD is predominantly derived from the Caucasian population, and studies involving the South Asian population are scant. In a review of novel pathogenic variants in the LAMA2 gene, only five (out of 64) patients of late-onset LAMA2-RD were reported, all belonging to Caucasian ethnicity, with the current variant not being described [13]. Since adult-onset LAMA2-RD is extremely rare, case reports of genetic variants, especially from ethnicities that may be underrepresented in genetic databases, are required to expand our understanding of the genetic and phenotypic spectrum of the disease. This is a single case report, and larger studies (especially prospective or retrospective cohort studies or case series) from non-Caucasian ethnicities may help unearth newer disease-causing variants in the LAMA2 gene and further update the genetic variant classification for this rare disease.

## Conclusions

To conclude, adult-onset limb-girdle muscular dystrophy with white matter imaging abnormalities, hyperCKemia, and seizures should evoke suspicion of LAMA2-RD. We report an extremely rare missense mutation causing late-onset LAMA2-RD in the South Asian population. More cases of late-onset LAMA2-RD from various ethnicities need to be reported to expand our understanding of the clinical-genetic spectrum of the disease. 
